# Neural responses when learning spatial and object sequencing tasks via imitation

**DOI:** 10.1371/journal.pone.0201619

**Published:** 2018-08-03

**Authors:** Elizabeth Renner, Jessica P. White, Antonia F. de C. Hamilton, Francys Subiaul

**Affiliations:** 1 Center for the Advanced Study of Human Paleobiology, The George Washington University, Washington, DC, United States of America; 2 Psychology, University of Stirling, Stirling, United Kingdom; 3 Department of Psychology, University of Nottingham, Nottingham, United Kingdom; 4 Institute of Cognitive Neuroscience, University College London, London, United Kingdom; 5 Department of Speech, Language, and Hearing Sciences, The George Washington University, Washington, DC, United States of America; 6 Smithsonian Institution, Washington, DC, United States of America; Ludwig-Maximilians-Universitat Munchen, GERMANY

## Abstract

Humans often learn new things via imitation. Here we draw on studies of imitation in children to characterise the brain system(s) involved in the imitation of different sequence types using functional magnetic resonance imaging. On each trial, healthy adult participants learned one of two rule types governing the sequencing of three pictures: a motor-spatial rule (in the spatial task) or an object-based rule (in the cognitive task). Sequences were learned via one of three demonstration types: a video of a hand selecting items in the sequence using a joystick (Hand condition), a computer display highlighting each item in order (Ghost condition), or a text-based demonstration of the sequence (Text condition). Participants then used a joystick to execute the learned sequence. Patterns of activation during demonstration observation suggest specialisation for object-based imitation in inferior frontal gyrus, specialisation for spatial sequences in anterior intraparietal sulcus (IPS), and a general preference for imitation in middle IPS. Adult behavioural performance contrasted with that of children in previous studies—indicating that they experienced more difficulty with the cognitive task—while neuroimaging results support the engagement of different neural regions when solving these tasks. Further study is needed on whether children’s differential performance is related to delayed IPS maturation.

## Introduction

Imitation, the ability to copy others’ responses, has intrigued scientists at least since Darwin linked imitation with human cognitive evolution [1]. Work in the developmental and comparative sciences has demonstrated that human imitation is distinct in its breadth (copying across tasks and domains), versatility (adaptively copying different responses), and fidelity (copying with high accuracy) [2,3]. These features are widely believed to allow people to imitate everything from dance moves to dress styles, from vocal accents to knowledge and expertise, preserving and perpetuating human culture.

While imitation of single, familiar actions has been widely studied, imitation of action sequences has received less attention. Studies of children’s behavioural performance in sequence imitation tasks suggest different developmental patterns for imitation of sequences defined by objects and imitation of sequences defined by actions to locations [4,5]. The aim of the present paper is to characterise the neural systems underlying these different types of sequence imitation learning. First, we review previous behavioural studies of sequence imitation, before turning to potential neural systems.

**Fig 1:**
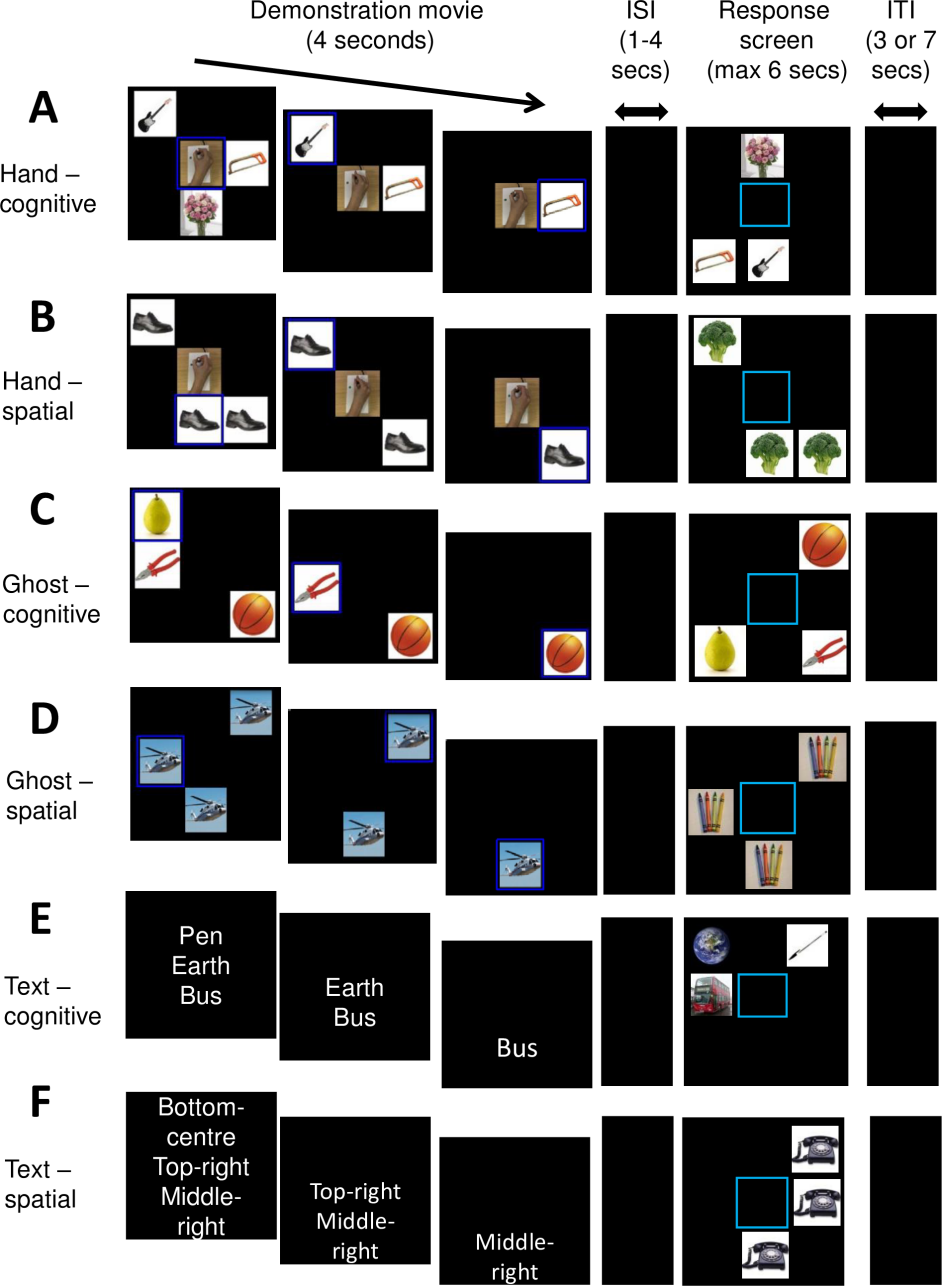
Sample sequences for each of the six trial types. Rows illustrate the following trial types: A, Hand-cognitive; B, Hand-spatial; C, Ghost-cognitive; D, Ghost-spatial; E, Text-cognitive; F, Text-spatial. Note that the demonstration phase differs between trial types while the response (execution) phases are very similar.

Our present project draws heavily on a detailed series of studies of how children [4–7] and non-human primates including monkeys [8,9] and orangutans [10] learn sequences on their own and from others. The two tasks used in this study, previously implemented in behavioural studies on touch-sensitive screens, test participants’ ability to learn and execute sequences governed by different underlying rules. In the ‘cognitive task’, individual objects must be selected in the correct order (e.g. apple—boy—cat) [11] regardless of the spatial locations of the objects. In the ‘spatial task’, items in specific locations must be selected in the correct order (e.g. top item—bottom item—right item) regardless of the identities of the items [4] (see Fig 1). The tasks are matched in terms of the number of items that must be attended to, encoded, and recalled, as well as in terms of motor responses (i.e., three touches are made to a screen). The use of sequences, rather than single items, ensures that high-accuracy performance can be attributed to attentiveness during a demonstration, rather than to chance.

For both tasks, the correct sequence can be learned in several different ways. If no instructions are available, participants can learn individually by trial and error. A demonstration from a knowledgeable person (presented live or via video) gives the opportunity to learn by imitation. A partially incorrect demonstration from another person can allow participants to infer the correct sequence. A verbal or written instruction cue could allow participants to perform correctly, but without any self-other matching. Finally, participants could learn by seeing the correct sequence of items highlighted on the computer without seeing another person, and matching the observed goals and results but not body or hand actions [12]; in this case, participants learn by emulation.

By using these sequencing tasks and various learning conditions, researchers have demonstrated that the imitation capacity does not develop in a concerted fashion but rather comes online unevenly in the preschool years [4,5,7]. That is, typically developing 3-year-old children can imitate sequences they see demonstrated by an experimenter in the cognitive task, but fail to imitate in the spatial task [4,5,13]. Follow-up studies and control conditions indicate that these dissociations between cognitive and spatial imitation are not due to difficulties learning spatial sequences in general, because children accurately recall spatial sequences they acquire by individual, trial-and-error, learning [4:Exp. 2, 13]. Moreover, 3-year-olds are able to infer a target sequence from a model’s errors in the spatial task, a result which demonstrates that they can learn spatial sequences vicariously [4:Exp. 3,7,13]. Around the age of 4, young children acquire the ability to learn sequences in the spatial task by watching an experimenter’s demonstration [4,7]. In sum, these results show that young children have a *specific* difficulty with imitating in the spatial task, which is independent of their sequencing performance in other conditions with both the spatial task and the cognitive task.

Both children and non-human primates (macaque monkeys) improve their performance on the cognitive task when given the opportunity to learn from a conspecific via imitation [8,9], compared to learning by trial-and-error. Additionally, both children and monkeys learn less well (or not at all) from ‘ghost’ demonstrations (see below) [6,8,14]. No dissociation between performance in the cognitive and spatial tasks has been found in non-human primates, although the spatial task has been less extensively used with these populations. One study with orangutans did not find clear differences between the tasks, but this may be attributable to poor overall performance [15].

The research presented here aims to explore the neural systems which underlie imitation performance in these tasks, potentially helping to shed light on the differential imitation behaviour observed in children. In particular, we aim to test whether imitation (as opposed to emulation or other forms of vicarious learning) in the cognitive and spatial tasks relies on different neural substrate(s). In this study, we adapt the cognitive and spatial sequencing tasks previously used with children and non-human primates [8–10] to be suitable for an fMRI environment. In every version of the sequencing tasks, participants must observe, remember, and then execute via joystick the selection of three items in a target sequence on a screen (Fig 1). The kind of information available differs during the demonstration phase of the task. We use three types of demonstration: the first involves learning from a human agent (Hand demonstration, Fig 1A and 1B). The second involves learning without a human agent, where the computer highlights the target items in order (Ghost or ‘no agent’ condition, Fig 1C and 1D). The term ‘ghost demonstration’ is derived from an extensive developmental literature showing that children copy events without a visible actor less robustly than those with a visible actor (reviewed in [12]). The third condition involves learning from reading text. In the Text condition, participants read written instructions for the target sequence (Fig 1E and 1F), which serves as a control condition for linguistic encoding and working memory. All three conditions make the same demands on working memory and sequencing, but differ in how information can be acquired.

**Fig 2:**
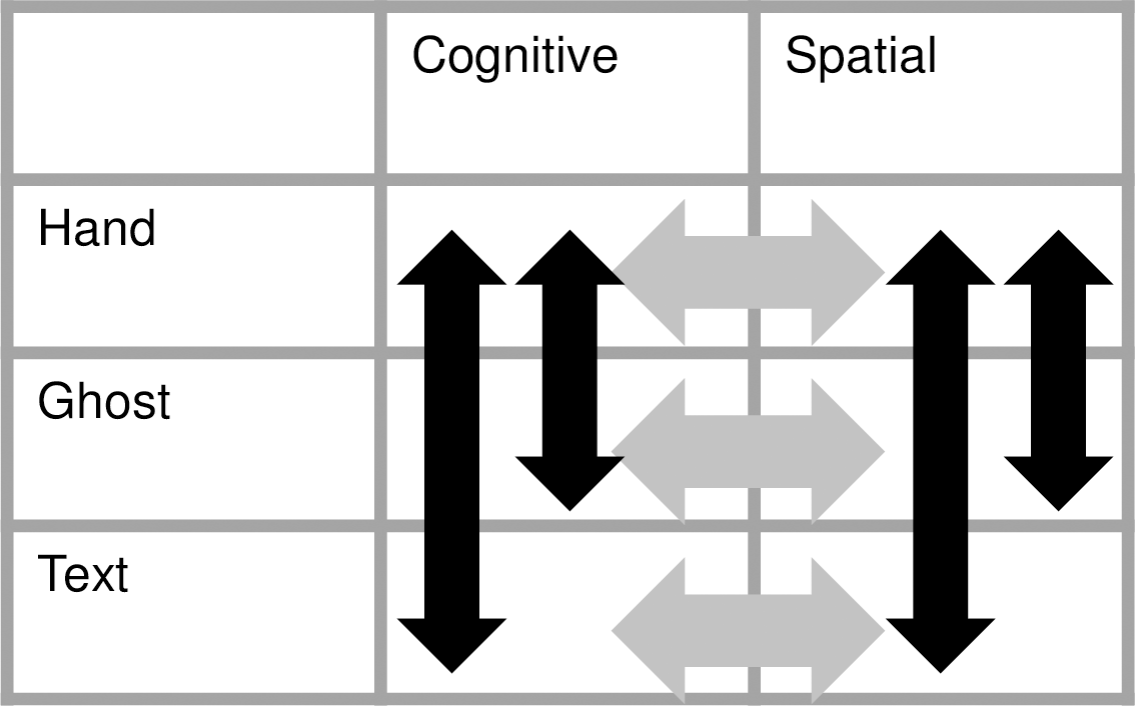
The full 3 x 2 factorial design. Arrows show each of the simple effects examined in the results.

Our overall design for the present study was a 2 × 3 within-subject factorial design, with the factors task (spatial/cognitive) and demonstration type (Hand/Ghost/Text), as illustrated in Fig 2. In this design, the Hand-cognitive (HC) and Hand-spatial (HS) conditions are the two conditions which allow imitation, where a participant sees hand actions and then performs corresponding hand actions. The other demonstration types provide control tasks without full imitation (such as emulation, in the Ghost condition). Our analysis in the present paper focuses on brain activations in the observation phase of these tasks when participants observe and learn a sequence with the intention of executing it. The execution phases were identical in all conditions. Analysis of the execution phase is presented elsewhere [Renner et al., in preparation].

To make predictions for which brain areas are likely to be engaged in our sequence learning tasks, we can draw on the wide range of past studies of action and imitation. Individual studies and meta-analyses indicate that parts of the ‘mirror neuron system’ are robustly activated during imitation tasks [16–18], although not every component is implicated in every task [17, 19–25]. We predicted that areas in the mirror neuron system—including inferior parietal and inferior frontal cortex—would be more activated by the Hand than the Text condition for both tasks. In addition, we predicted that the cognitive task might engage regions linked to object processing and identification in the occipital and temporal lobes [26,27], while the spatial task might engage regions linked to spatial processing in the superior parietal lobe [27,28]. Finally, if there are neural systems which are specific to particular types of imitation, as suggested by the developmental data [4,5,7,13], then there may be robust differences between the spatial and cognitive tasks as well as between the Hand and Text conditions, or interactions between these conditions. That is, differences should emerge in both the horizontal and vertical contrasts in Fig 2, as well as interactions. The precise pattern of the contrasts will provide insight into which neural systems have a role in the different types of imitation which we study.

## Materials and methods

### Participants

Nineteen right-handed adults (10 female; median age 20 years) were recruited via local publicity. Four additional adults took part in the study but were excluded from analysis due to completion of less than two full scanning runs or excessive errors (>30% of trials). Written informed consent was obtained from all participants before testing. All procedures were approved by the University of Nottingham Medical School ethics committee.

### Tasks

Two touchscreen-based tasks used previously with typically developing children and children with autism [4,5,7,9,14] and with non-human primates [8,10,11,29] were adapted so that participants could respond with a joystick in an fMRI scanner.

*Cognitive Task* [11] (Fig 1A, 1C and 1E). In the cognitive task, images of three different easily distinguishable objects appear simultaneously on a screen. Participants must select images in a specific order, independently of the positions of the images on the screen; the spatial arrangement of the pictures varies randomly between the observation and execution phases. This feature prevents participants from learning the sequence via a motor response. Instead, participants must form an abstract ‘cognitive’ representation (based on the content of the pictures) rather than a spatial representation to respond correctly to the items. The sequence illustrated in Fig 1A is flowers → guitar → saw. Without demonstration, the chance of correctly responding to a three-item sequence is 16.67% (i.e., 1/3 [33%] × 1/2 [50%] × 1/1 [100%] = 16.67%).

*Spatial Task* [4] (Fig 1B, 1D and 1F). This task is in most respects exactly like the cognitive task described above, with the following exceptions: (i) all of the images are identical within a single trial, (ii) the identity of the images changes between observation and execution, and (iii) the spatial configuration of the images remains fixed. These features of the task require that participants respond on the basis of spatial location, rather than object identity (in contrast to the cognitive task). The sequence illustrated in Fig 1D is middle-left → top-right → bottom-centre. Similarly to the cognitive task, the chance of correctly responding to a three-item sequence on the first trial (without demonstration) is 16.67%.

These two tasks were presented with three different types of demonstration. In a Hand demonstration, the centre of the screen shows a video of a hand moving an fMRI-compatible joystick, and pictures are present in 3 of 8 possible grid locations surrounding the centre video. As the hand moves the joystick, the picture in the corresponding location is selected and a blue border appears around the picture. The picture then disappears to indicate that it has been correctly selected (Fig 1A and 1B). A Ghost demonstration is the same as above for the Hand demonstration, except that the centre of the screen remains blank. As in the Hand demonstration, each image is highlighted in the correct order, with a blue border appearing around each image and images disappearing once selected (Fig 1C and 1D). In a Text demonstration, the correct sequence is written in English words which disappear one at a time with the same timing as the pictures in the Hand and Ghost demonstrations (Fig 1E and 1F). Together, the two tasks and three demonstration types give a within-subject 2 × 3 factorial design for this study. Conditions from this design are illustrated in Fig 2.

### Task implementation

Each trial comprised two phases: an observation phase of 4 seconds and an execution phase of 6 seconds. An interval randomly selected from between 1 and 4 seconds was imposed between these phases. An inter-trial interval of 3 seconds or 7 seconds separated successive trials.

Observation-phase stimuli for the Hand demonstration were generated by recording video clips of a human hand on an MRI-compatible joystick while an actor selected three images on the screen by moving the joystick in turn to 3 of 8 cardinal directions. Matlab-based video editing scripts were used to place these hand action clips in the centre of a video depicting three items which were selected and then removed from the screen one at a time (see Fig 1). Items were drawn from a set of 100 images of nameable objects. For the Ghost demonstration trials, equivalent clips without the hand video were generated (Fig 1C and 1D). For the Text demonstration trials, video clips showed three object names which disappeared in turn (Fig 1E and 1F). All demonstration clips were 4 seconds long.

A black screen was shown during the inter-stimulus interval. Then the response screen of the execution phase appeared; it showed either the same three pictures shuffled into a different spatial configuration (for the cognitive task) or a different set of three identical pictures located in the same spatial configuration as in the demonstration (for the spatial task). Participants responded by using an fMRI-compatible joystick (Current Designs, Philadelphia, PA) identical to the one appearing in the demonstration videos. As the participant moved the joystick, a blue square highlighted the image selected, and the image disappeared to indicate that it had been correctly selected. The task was implemented using custom-written Cogent scripts in Matlab.

Participants received feedback on their performance for each trial. When each picture was correctly selected, it disappeared; therefore, correct selection of all three pictures in the sequence resulted in the disappearance of all pictures. Two types of error were possible: incorrect picture selection and slow response. When an incorrect picture was selected, the trial ended and the word ‘Error’ appeared on the screen until the start of the next trial. If the correct sequence was not completed in the allotted time (6 seconds), the word ‘Error’ appeared briefly on the screen before the start of the next trial. Before beginning the study, participants were told to perform as quickly and accurately as possible and to minimise errors.

### fMRI scanning

Participants performed 48 practice trials in the scanner to become familiar with the tasks. A T1 anatomical scan was collected during this time. After the practice trials, two sessions of 48 experimental trials each were conducted, for a total of 96 experimental trials per participant. Trials were drawn from a 2 × 3 factorial design with 8 trials in each cell for the factors task (cognitive/spatial) and demonstration type (Hand/Ghost/Text). Trial order within each session was pseudorandomised. Pseudorandom trial order (different for each participant) was generated by permuting the complete list of possible trials.

For each session, 308 images were collected in a 3T Phillips scanner using double echo imaging, with 37 slices per TR (3 mm thickness) and the following settings: TR, 2500 ms; TE, 20 and 45 ms; flip angle, 80°; field of view, 19.2 cm; matrix, 64 × 64. Double echo imaging was used to improve signal detection [30], and the two images were combined using a weighted summation based on the signal strength in each brain region [31].

### Data analysis

Preprocessing steps included realignment and unwarping; normalisation to the standard SPM EPI template; and smoothing by 8 mm in SPM8 (The Wellcome Department of Imaging Neuroscience, London, UK; http://www.fil.ion.ucl.ac.uk/spm/software/) in Matlab 2012a (The MathWorks, Inc., Natick, MA). After preprocessing, quality control measures included a registration check (to ensure that after preprocessing, brains were correctly aligned) and a check for excessive head movement. If not mentioned otherwise, SPM default parameters were used.

A design matrix was fitted for each participant. The matrix modelled the observation events according to their category (see Fig 2). Each observation event had a 4-second duration. Execution events were modelled in a similar fashion, with a 6-second duration for each. If the participant made an error, both the observation and execution phase associated with that error were modelled in separate ‘demo-error’ and ‘response-error’ categories. Thus, there were 14 regressors for each of the two sessions (S1 Table).

### Statistical analysis

Contrasts at the second level were calculated separately for the observation and execution phases, and we focus here on the observation phase only. We examine simple effects (the effect of one independent variable within a single level of another independent variable) rather than main effects, because this allows us to identify overlap between different tasks and demonstration types (see Fig 2 for a summary of the simple effects calculated). To localise brain regions specific to the cognitive task, we examine the simple effects of task (cognitive > spatial) for each of the three demonstration types (Hand, Ghost, and Text): specifically, we examined the contrasts Hand-cognitive > Hand-spatial, Ghost-cognitive > Ghost-spatial, and Text-cognitive > Text-spatial. To localise brain regions specific to the spatial task, we examine the inverse simple effects (spatial > cognitive) for each demonstration type: specifically, Hand-spatial > Hand-cognitive, Ghost-spatial > Ghost-cognitive, and Text-spatial > Text-cognitive. Each simple effect was thresholded at *p*<0.001 uncorrected and 50 voxels, which is equivalent to a familywise error threshold of *p*<0.055. We also consider the overlap of these simple effects of demonstration type. If the simple effects overlap, this indicates an activation that is specific to a particular task but not sensitive to demonstration type.

We also test for general ‘imitation’ regions by calculating the simple effects of demonstration type (Hand > Text) for the cognitive task and for the spatial task separately. The same threshold was used. If these simple effects overlap, the regions of overlap may provide a general imitation system. We can test for differences between the two vicarious learning conditions (Hand vs. Ghost condition) with the simple effects of Hand > Ghost for the cognitive task and for the spatial task separately. Finally, we test for the following interactions: [Hand-cognitive–Hand-spatial] > [Ghost-cognitive–Ghost-spatial]; [Hand-cognitive − Hand-spatial] > [Text-cognitive − Text-spatial]; and [Ghost-cognitive–Ghost-spatial] > [Text-cognitive–Text-spatial]. Each interaction was calculated with a *t* test in SPM, and both the positive and negative effects were examined. For all these contrasts, we thresholded our images at *p*<0.001 uncorrected with 50 voxels, equivalent to a familywise error threshold of *p*<0.055.

A behavioural analysis of response times (the time it took to select all three images) and error rates was performed, and results were compared via 2 × 3 (task type × demonstration type) ANOVA in SPSS (version 21.0, IBM, Armonk, NY). Data from trials in which errors were made were not included in the behavioural analyses.

## Results

### Behavioural results

**Fig 3:**
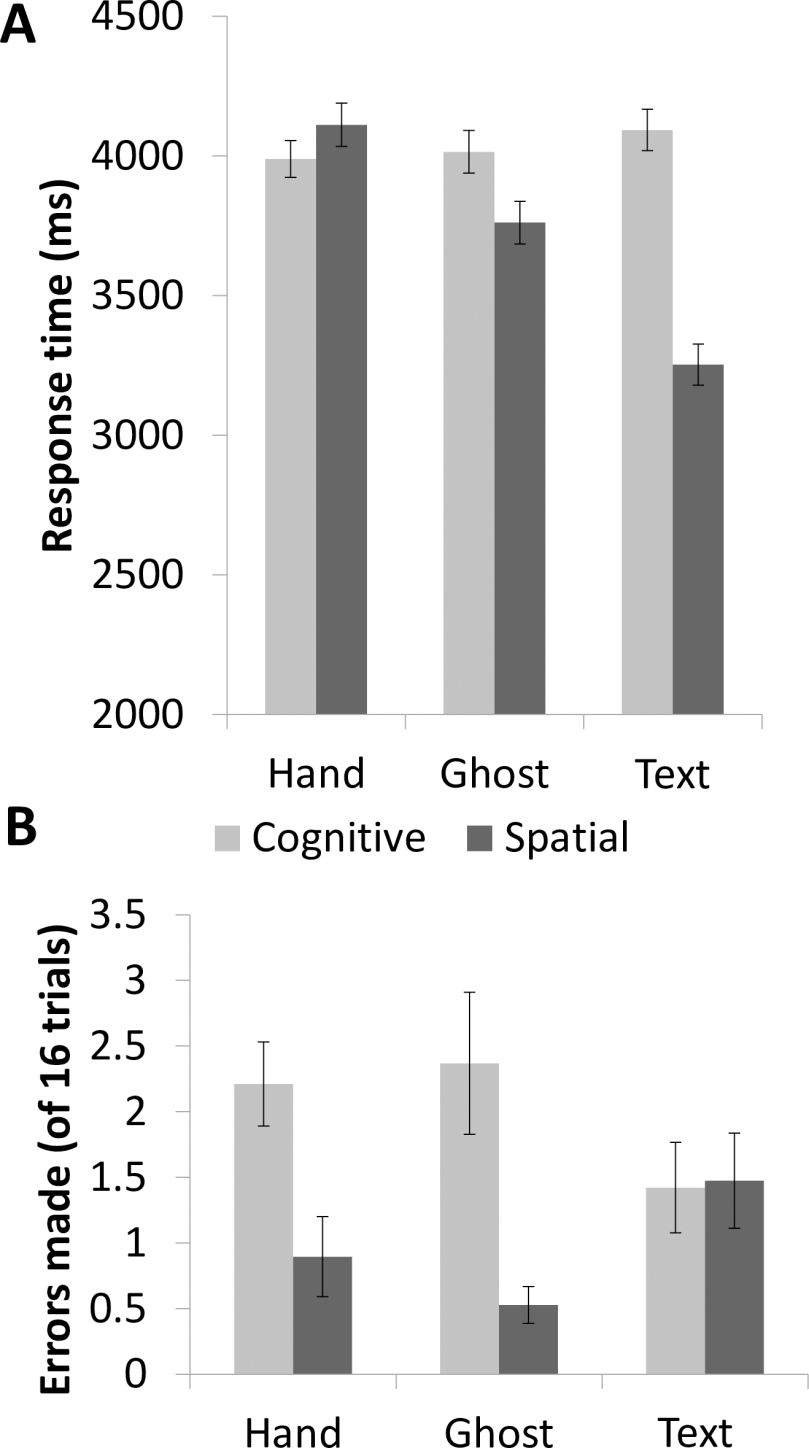
**Reaction times (A) and error rates (B) when participants performed the task during fMRI.** Error bars show standard errors.

Response times showed task-based as well as demonstration-based differences (Fig 3A). A repeated-measures ANOVA with task type (cognitive and spatial) and demonstration type (Hand, Ghost, and Text) as within-subjects factors indicated that there were significant main effects of both task type [*F*(1,18) = 64.77, *p* < 0.001] and demonstration type [*F*(2,36) = 30.81, *p* < 0.001], as well as a significant interaction between task type and demonstration type [*F*(2,36) = 49.58, *p* < 0.001]. A one-way repeated-measures ANOVA to test for simple effects showed that response times after different demonstrations for the cognitive task were not significantly different from each other [*F*(2,36) = 0.881, *p* = 0.423, partial η^2^ = 0.047]. However, response times after different demonstrations for the spatial task differed significantly [*F*(2,26.008) = 134.032, *p* < 0.001, partial η^2^ = 0.882; Mauchly’s test of sphericity showed that the assumption of sphericity had been violated, Χ^2^(2) = 8.243, *p* = 0.016, so the Greenhouse-Geisser correction for degrees of freedom was used (ε = 0.722)]. Response times after Hand demonstrations of the spatial task (mean = 4,112 ms) were slower than those after Ghost demonstrations (mean = 3,761 ms), and those after Ghost demonstrations were slower than those after Text demonstrations (mean = 3,253 ms), *F*(2,36) = 134.032, *p* < 0.001, partial η^2^ = 0.882.

For error rates (Fig 3B), a repeated-measures ANOVA with task type (cognitive and spatial) and demonstration type (Hand, Ghost, and Text) as within-subjects factors indicated a significant main effect of task type [*F*(1,18) = 25.02, *p* < 0.001] but not of demonstration type, and a significant interaction between task type and demonstration type [*F*(2,36) = 4.20, *p* = 0.023]. A one-way repeated-measures ANOVA to test for simple effects showed that error rates after different demonstrations of the cognitive task were not significantly different [*F*(2,36) = 2.396, *p* = 0.105]; error rates also did not differ after the different demonstrations of the spatial task [*F*(1.305,36) = 3.441, *p* = 0.066; Mauchly’s test of sphericity showed that the assumption of sphericity had been violated, Χ^2^(2) = 12.920, *p* = 0.002, so the Greenhouse-Geisser correction for degrees of freedom was used (ε = 0.653)]. However, one-way ANOVAs found that significantly more errors were made for the cognitive task than the spatial task following both Hand [*F*(1,18) = 12.309, *p* = 0.003, partial η^2^ = 0.406] and Ghost demonstrations [*F*(1,18) = 16.455, *p* = 0.001, partial η^2^ = 0.478]. There was no difference between errors made in the cognitive and the spatial tasks following Text demonstrations [*F*(1,18) = 0.012, *p* = 0.915]. Error trials were removed from fMRI analysis so differences in error rates cannot impact on the fMRI results.

### Imaging results: Effects of task

**Fig 4:**
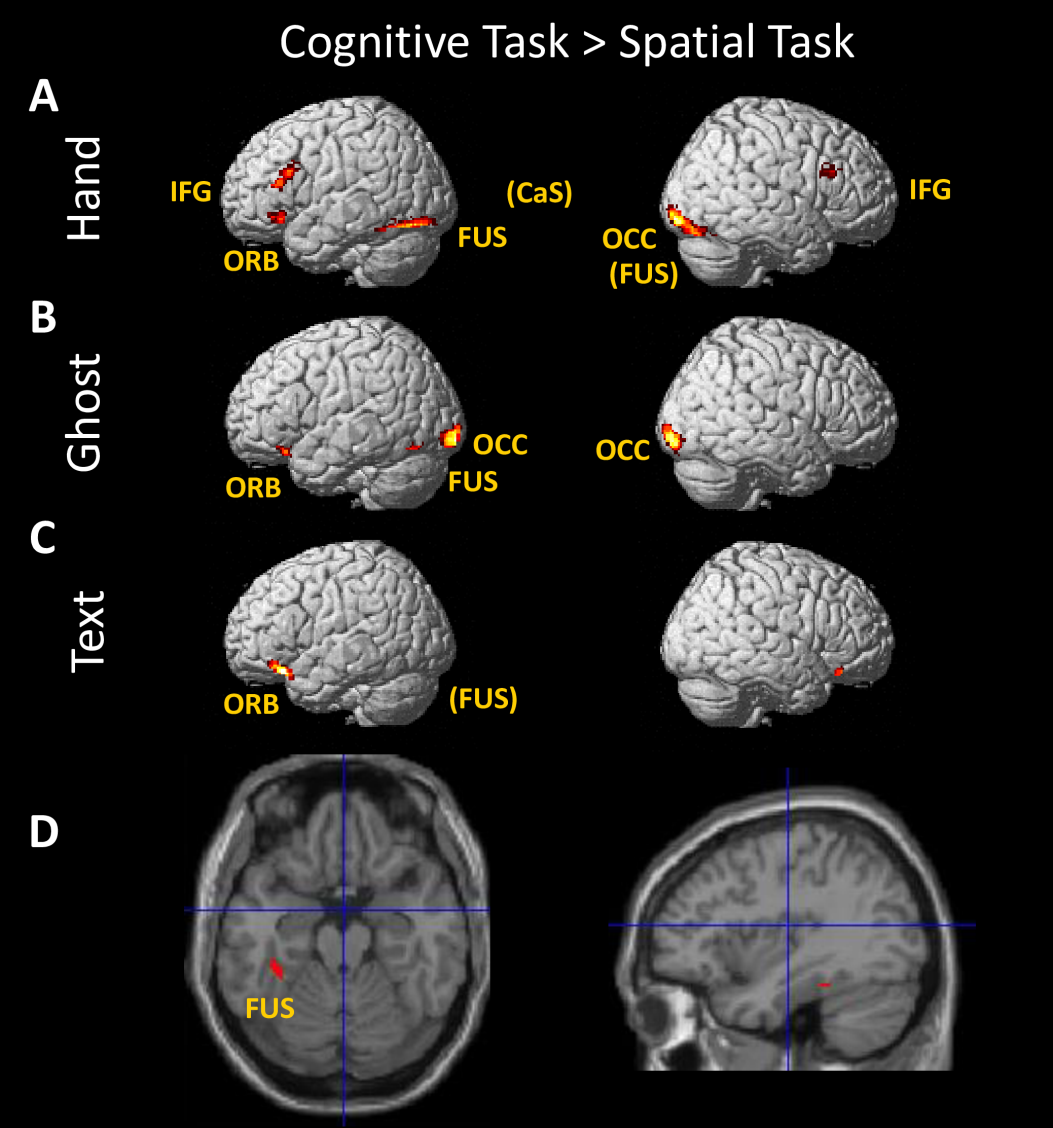
**Simple effects of viewing demonstrations of the cognitive task compared to the spatial task for each of the three demonstration types: Hand (A), Ghost (B), and Text (C).** (D) Conjunction of the three contrasts shown above. The coordinates of the area activated in all three conditions are −35 −36 −20. Coordinates for all clusters are given in Table 1. Abbreviations: CaS, calcarine sulcus; FUS, fusiform gyrus; IFG, inferior frontal gyrus; OCC, occipital gyrus; ORB, orbital gyrus. Region names in parentheses indicate that clusters are not visible in this view.

Greater brain activation when observing demonstrations of the cognitive task compared to the spatial task was found in left fusiform gyrus across all three demonstration types in a conjunction analysis (Fig 4D). Both Hand and Ghost conditions engaged lateral occipital regions and left orbital gyrus during the cognitive task (Fig 4A and 4B). In the Hand condition only, there was greater activation of bilateral inferior frontal gyrus for the cognitive task than the spatial task. In the Text condition only, there was greater activation of the bilateral cingulate gyrus and left posterior orbital gyrus. All of these effects are illustrated in Fig 4 and detailed in Table 1.

**Table 1:** Brain region, MNI coordinates, size (no. of voxels, k), and significance of clusters.

Region	Details for Hand					Details for Ghost					Details for Text				
	x	y	z	k	p(FWE)	x	y	z	k	p(FWE)	x	y	z	k	p(FWE)
**Cognitive > Spatial**	** **				** **	** **				** **	** **				
Left fusiform gyrus	-28	-58	-18	647	<0.001	-30	-58	-14	172	<0.001	-36	-28	-22	72	0.040
Right fusiform gyrus	36	-40	-24	91	0.003										
Right inferior occipital gyrus	34	-96	-8	438	<0.001	28	-100	-4	271	<0.001					
Left inferior occipital gyrus						-18	-102	-10	244	<0.001					
Bilateral calcarine sulcus	14	-80	8	628	<0.001										
Left inferior frontal gyrus	-42	28	16	208	<0.001										
Left inferior frontal gyrus	-46	28	-10	73	0.010										
Right inferior frontal gyrus	40	20	26	77	0.007										
Left orbital gyrus	-20	28	-14	55	0.041	-30	26	-18	76	0.018					
Left posterior orbital gyrus											-38	24	-20	274	<0.001
Bilateral cingulate gyrus											-6	30	-10	670	<0.001

**Fig 5:**
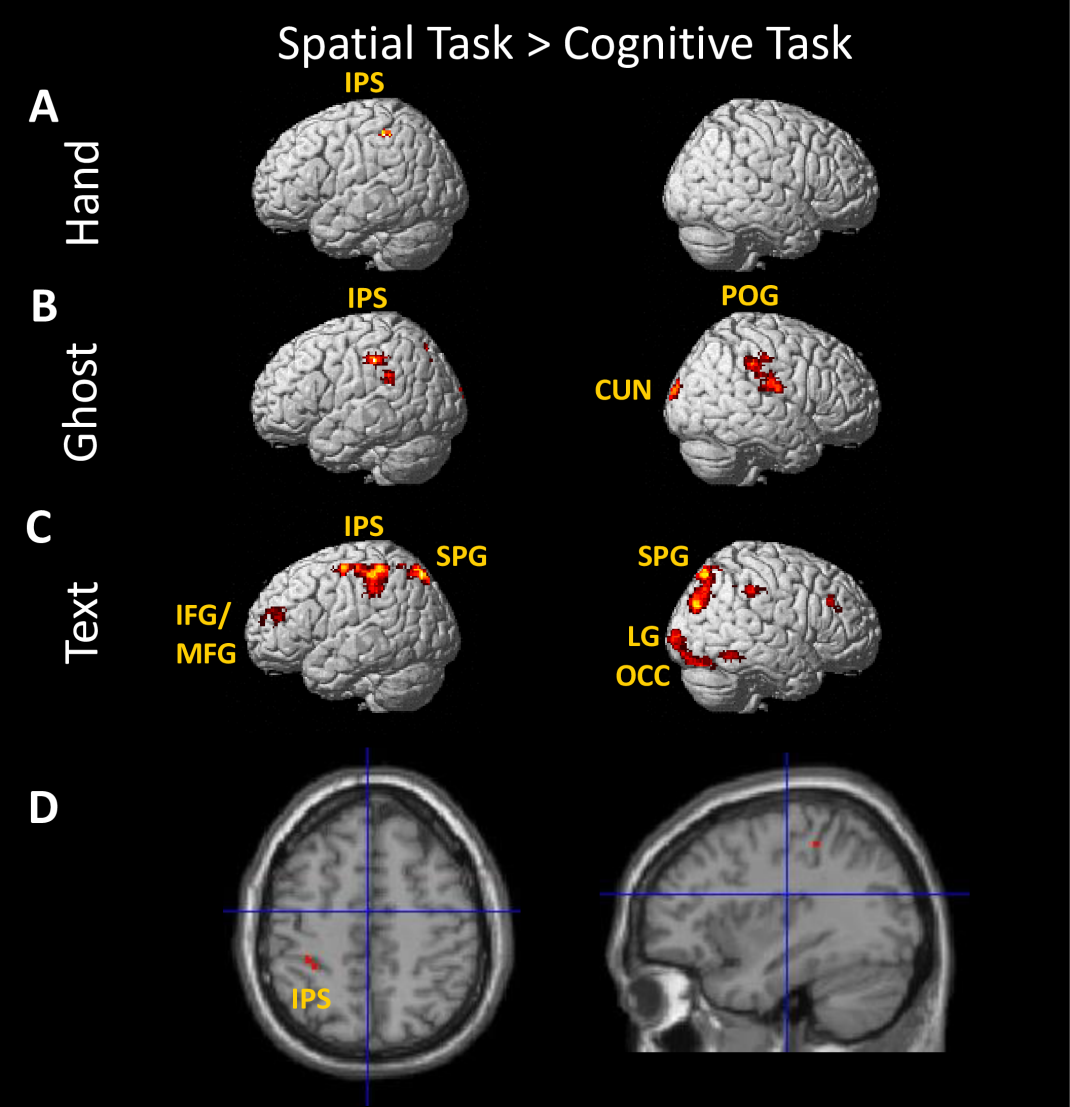
**Simple effects of viewing demonstrations of the spatial task compared to the cognitive task for each of the three demonstration types: Hand (A), Ghost (B), and Text (C).** (D) Conjunction of the three contrasts shown above. The coordinates of the area activated in all three conditions are −35 −36 50. Coordinates for all clusters are given in Table 2. Abbreviations: CUN, cuneus; IFG/MFG, inferior frontal gyrus/middle frontal gyrus; IPS, intraparietal sulcus; LG, lingual gyrus; OCC, occipital gyrus; PCUN, precuneus; POG, postcentral gyrus; SPG, superior parietal gyrus. Clusters of activation in the precuneus (Ghost) and right caudate nucleus, left putamen, and right cerebellum (Text) are not visible in this depiction.

Greater brain activation when observing demonstrations of the spatial task compared to the cognitive task was found in the left intraparietal sulcus across all three demonstration types in a conjunction analysis (Fig 5D). In the Hand condition, there were no other significant activations (Fig 5A). In the Ghost condition, there was also activation of right postcentral gyrus, bilateral precuneus, a second area in left intraparietal sulcus, and right cuneus for the spatial task compared to the cognitive task (Fig 5B). In the Text condition, there was greater activation of right cerebellum, right caudate nucleus, bilateral superior parietal gyrus, left putamen, left inferior/middle frontal gyrus, and right inferior occipital gyrus for the spatial task than the cognitive task (Fig 5C). These effects are illustrated in Fig 5 and detailed in Table 2.

**Table 2:** Brain region, MNI coordinates, size (no. of voxels, k), and significance of clusters.

Region	Details for Hand					Details for Ghost					Details for Text				
	x	y	z	k	p(FWE)	x	y	z	k	p(FWE)	x	y	z	k	p(FWE)
**Spatial > Cognitive**															
Right postcentral gyrus						64	-12	22	333	<0.001					
Right postcentral gyrus						36	-34	22	67	0.033					
Bilateral precuneus						-12	-76	36	386	<0.001					
Right cuneus						14	-100	16	137	<0.001					
Left intraparietal sulcus						-28	-40	48	299	<0.001					
Left intraparietal sulcus	-34	-38	54	52	0.053	-44	-44	28	101	0.004	-36	-34	52	1261	<0.001
Bilateral superior parietal gyrus											-16	-74	54	2447	<0.001
Right lingual gyrus											14	-94	-6	682	<0.001
Right caudate nucleus											18	-14	22	157	<0.001
Right caudate nucleus											20	22	6	78	0.028
Left putamen											-20	4	8	160	<0.001
Left inferior/middle frontal gyrus											-44	44	18	95	0.010
Right cerebellum											0	-56	-18	133	0.001
Right cerebellum											20	-56	-22	96	0.010
Right inferior occipital gyrus											60	-54	-14	78	0.028

### Imaging results: Effects of demonstration type

**Fig 6:**
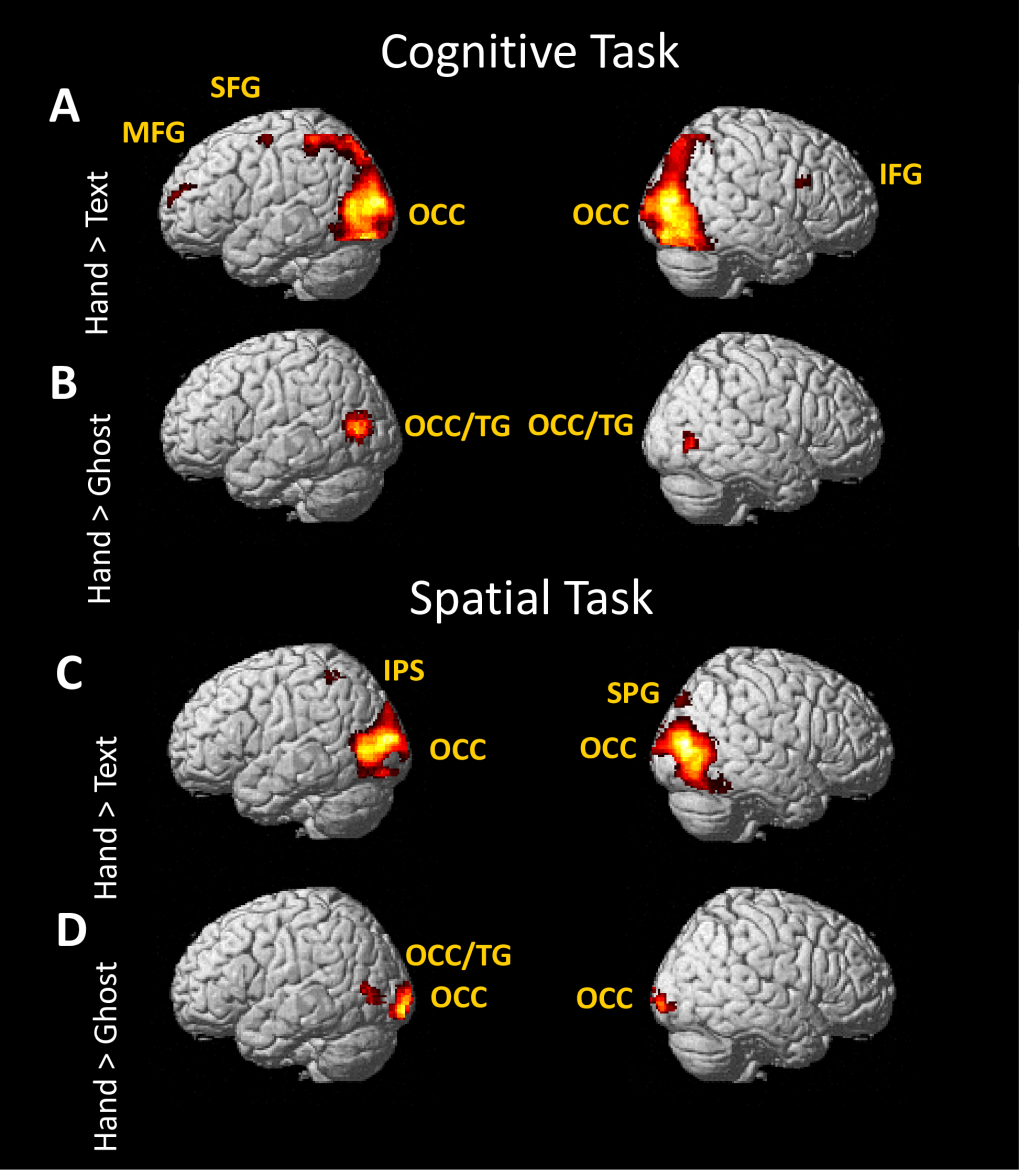
**Specialisation for observing hand actions in the cognitive task (A and B) and the spatial task (C and D).** IFG, inferior frontal gyrus, IPS, intraparietal sulcus; MFG, middle frontal gyrus; OCC, occipital gyrus; SFG, superior frontal gyrus; SPG; superior parietal gyrus; TG, temporal gyrus. Clusters of activation in the thalamus (A) and precuneus (C) are not visible in this depiction.

Observing Hand demonstrations compared to observing Text demonstrations resulted in a substantial activation across lateral occipitotemporal cortex in both the cognitive and spatial tasks (Fig 6A and 6C). In the cognitive task, Hand demonstrations compared to Text demonstrations also engaged left superior and middle frontal gyrus, right inferior frontal gyrus, and right thalamus. In the spatial task, Hand demonstrations compared to Text demonstrations engaged left intraparietal sulcus, left precuneus, and right superior parietal gyrus (Table 3).

**Table 3:** Brain region, MNI coordinates, size (no. of voxels, k), and significance of clusters showing activation in the Hand or Text condition.

Region	Details for Cognitive					Details for Spatial				
	x	y	z	k	p(FWE)	x	y	z	k	p(FWE)
**Hand > Ghost**										
Left occipital gyrus/temporal gyrus	-54	-74	10	419	<0.001	-50	-70	6	117	0.002
Left occipital gyrus						-22	-100	-8	414	<0.001
Right occipital gyrus/temporal gyrus	56	-70	0	115	<0.001					
Right occipital gyrus						26	-100	-6	155	<0.001
**Hand > Text**										
Bilateral occipital gyri extending into MTG^a^	30	-84	-14	14987	<0.001					
Right middle occipital gyrus						40	-80	2	4353	<0.001
Left middle occipital gyrus						-26	-96	14	3251	<0.001
Right thalamus	16	-14	18	180	<0.001					
Left superior frontal gyrus	-24	-8	58	164	<0.001					
Left middle frontal gyrus	-26	64	14	77	0.008					
Right inferior frontal gyrus	42	16	26	69	0.015					
Right superior parietal gyrus						20	-86	46	86	0.012
Left precuneus						-12	-54	22	69	0.034
Left intraparietal sulcus						-34	-46	56	63	0.051
**Text > Hand**										
Midline cuneus and precuneus	2	-84	18	1151	<0.001	0	-86	38	2919	<0.001
Midline anterior cingulate gyrus	2	38	-6	190	<0.001					
Right supramarginal gyrus	60	-60	28	116	0.001					
Left angular gyrus	-48	-72	32	54	0.049					
Left middle/inferior frontal gyrus						-44	18	28	429	<0.001
Right middle/inferior frontal gyrus						40	24	24	89	0.010
Right caudate nucleus						18	-12	22	78	0.019

^a^, this area overlaps with the two listed below.

Observing Hand demonstrations in both the cognitive and spatial tasks, compared to Ghost demonstrations, led to activations in occipitotemporal cortex (Fig 6B and 6D). Left and right occipital gyri were also more engaged for Hand than Ghost spatial demonstrations.

### Imaging results: Overlaps and interactions

**Fig 7:**
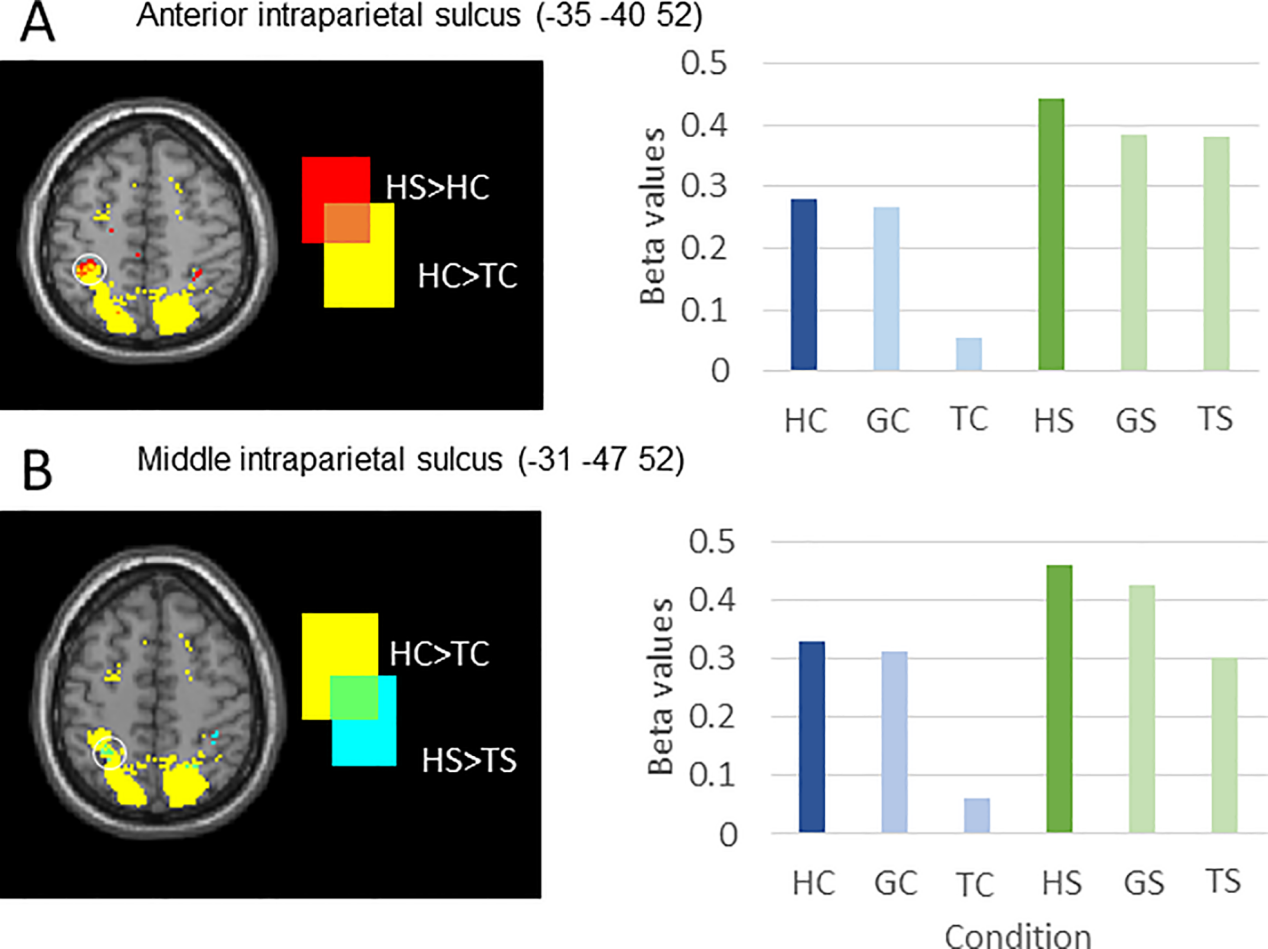
Detail of effects in intraparietal sulcus. HC, Hand-cognitive; GC, Ghost-cognitive; TC, Text-cognitive; HS, Hand-spatial; GS, Ghost-spatial; TS, Text-spatial. (A) An anterior region of left IPS shows a significant pattern HS > HC and HC > TC, that is, this region responds preferentially to a Hand demonstration of the spatial task. (B) Middle intraparietal sulcus responds selectively to Hand demonstrations over Text demonstrations in both the cogntive task and the spatial task. Parameter estimates (betas) for the six conditions in the overlapping areas are shown in the bar graphs (right).

Regions in the parietal cortex were engaged in several of the contrasts described above. To understand these patterns of overlap, we plotted different combinations of contrasts on the same brain image, and examined the parameter estimates (beta values) in regions where contrasts overlapped. We found that a region of the left anterior intraparietal sulcus (MNI coordinate −35 −40 52) was more active for the Hand-spatial task than the Hand-cognitive task, and was also more active for the Hand-cognitive task than for the Text-cognitive task (Fig 7A). A region in the left middle intraparietal sulcus (MNI coordinate −31 −47 52) was more active for Hand demonstrations than Text demonstrations of both the cognitive and the spatial task.

**Fig 8:**
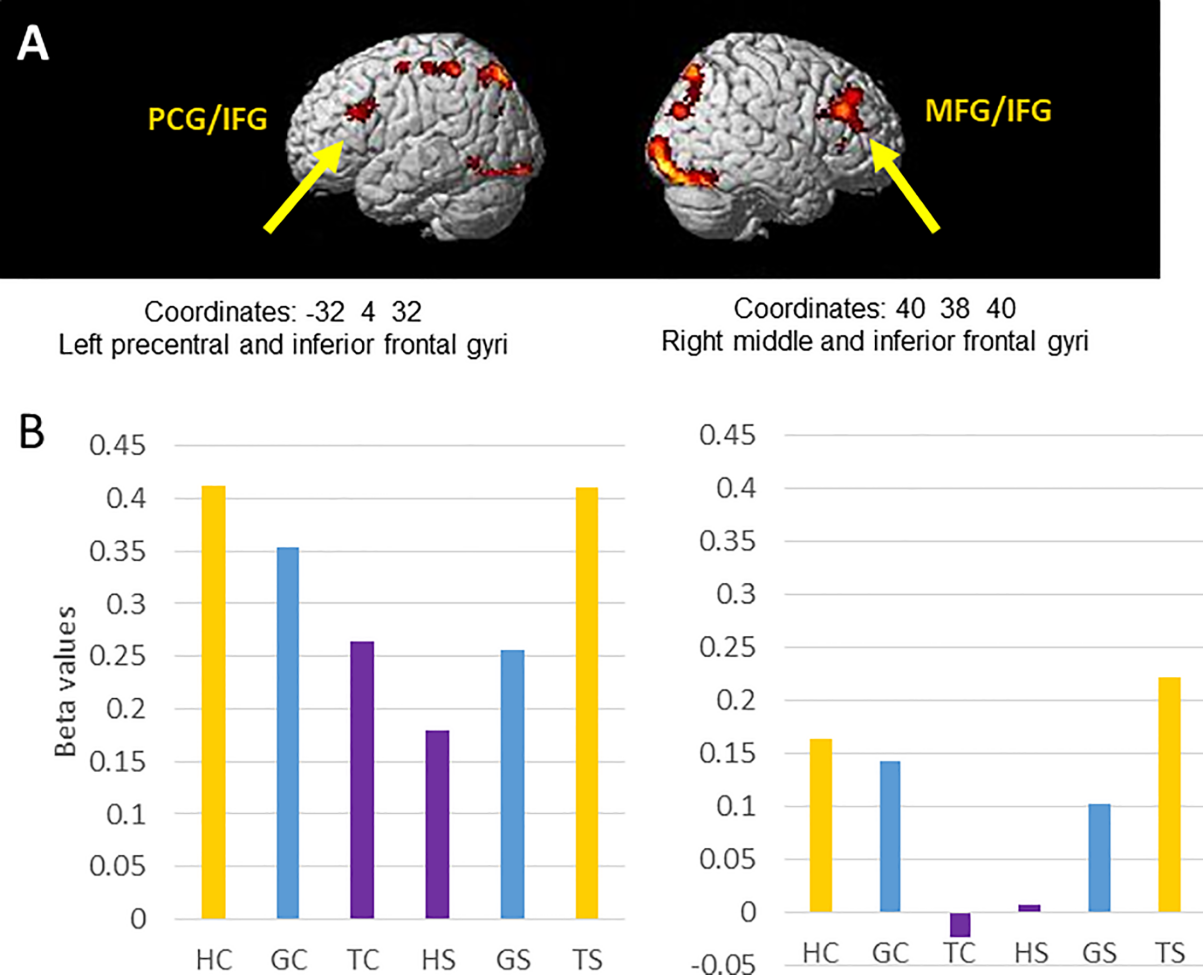
Some results from the interaction contrast [Hand-cognitive–Hand-spatial] > [Text-cognitive–Text-spatial]. Two clusters are illustrated here, which include left precentral and inferior frontal gyri and right middle and inferior frontal gyri. (A) Visualisation of the significantly activated clusters in this contrast. (B) Parameter estimates (betas) for the two clusters. Orange bars indicate conditions weighted positively in this interaction, purple bars indicate conditions weighted negatively, and blue bars indicate conditions that were not evaluated in this contrast. Coordinates for all clusters activated in this condition are given in Table 4. Abbreviations: IFG, inferior frontal gyrus; MFG, middle frontal gyrus; PCG, precentral gyrus.

Three interaction contrasts were tested, each in both positive and negative directions, and full results of all interactions are given in Table 4. We highlight the finding that the interaction [Hand-cognitive − Hand-spatial] > [Text-cognitive − Text-spatial] showed a bilateral activation of inferior frontal gyrus. This effect is shown in Fig 8, with the parameter estimates (betas) plotted for illustration. The plots suggest that the interaction is driven by a stronger activation in the Hand condition of the cognitive task compared to the spatial task.

**Table 4:** Brain region, MNI coordinates, size (no. of voxels, k), and significance of clusters activated in the interaction contrasts.

Region(s) differentially activated	Details				
	x	y	z	k	p(FWE)
**Demo [Hand Cog—Hand Spa] > [Ghost Cog—Ghost Spa]**					
Right superior occipital gyrus	14	-94	28	186	<0.001
Left lingual gyrus	-10	-84	-10	148	<0.001
**Demo [Hand Spa—Hand Cog] > [Ghost Spa—Ghost Cog]**					
No significant clusters					
**Demo [Hand Cog—Hand Spa] > [Text Cog—Text Spa]**					
Right fusiform and occipital gyrus extending to cerebellum	30	-68	-24	1861	<0.001
Large area in bilateral superior parietal lobe	-12	-76	50	1589	<0.001
Right middle and inferior frontal gyri	40	38	40	504	<0.001
Left precentral and inferior frontal gyri	-32	4	32	253	<0.001
Left fusiform, fourth occipital, & inferior occipital gyri	-36	-70	-16	251	<0.001
Right thalamus	14	-18	16	229	<0.001
Left postcentral gyrus/supplementary motor area	-38	-42	60	171	<0.001
Left lingual gyrus	-10	-88	2	126	0.001
Left precentral gyrus	-28	-4	56	92	0.004
Left angular gyrus	-26	-68	32	61	0.038
**Demo [Hand Spa—Hand Cog] > [Text Spa—Text Cog]**					
Left anterior cingulate cortex	-4	38	-8	341	<0.001
Left angular gyrus	-50	-70	32	57	0.052
**Demo [Ghost Cog—Ghost Spa] > [Text Cog—Text Spa]**					
Right middle and inferior occipital gyri	34	-96	-10	875	<0.001
Left inferior occipital gyrus	-28	-92	-12	325	<0.001
Right superior parietal gyrus	24	-64	50	306	<0.001
Right anterior insula	36	26	4	191	<0.001
Left superior parietal gyrus	-14	-72	56	127	0.001
**Demo [Ghost Spa—Ghost Cog] > [Text Spa—Text Cog]**					
Bilateral cingulate gyrus	-6	32	0	399	<0.001
Left precuneus	-16	-50	34	204	<0.001

Abbreviations: Cog, cognitive task; Spa, spatial task.

## Discussion

While many neuroimaging studies have explored the imitation of single, familiar actions such as the lifting of a finger or the grasping of objects [16], few studies have examined the imitation of action sequences. Using standardised tasks and procedures from the developmental sciences, the present study sought to identify the neural systems involved in vicariously learning novel sequences across different conditions and tasks in adults. The results show activations within the predicted brain network, which includes the inferior frontal gyrus, intraparietal sulcus, and occipitotemporal cortex.

### Differences between cognitive and spatial tasks

Previous studies pointed to different developmental trajectories for imitation in the cognitive and spatial tasks, and one of our aims in this study was to determine if there are different neural systems underlying learning in these tasks. Stronger activation for the cognitive task compared to the spatial task was found in both left fusiform gyrus and left orbital gyrus for all three demonstration types (Fig 4 and Table 1). This activation pattern suggests that these areas have a general role in recognising and responding to object sequences, but are not dedicated to imitative actions per se, because they respond to all three demonstration types. Stronger activation for the spatial task than the cognitive task was found in left anterior IPS (Figs 5D and 7A). This area shows a stronger response to the spatial task compared to its cognitive counterpart with each type of demonstration (Hand-spatial > Hand-cognitive, Ghost-spatial > Ghost-cognitive, Text-spatial > Text-cognitive), indicating strong selectivity for the spatial task. This activation pattern suggests that this area responds to spatial patterns, but is not specifically activated when observing to imitate, because it responds to all three demonstration types.

To link these areas to specific cognitive components would require reverse inference, which is outside the scope of this paper. However, we note that previous work shows that object recognition and memory tasks, especially those that use pictorial material, engage fusiform cortex [32]. In addition, various visuospatial working memory tasks engage parietal cortex [27, 33–37], including inferior parietal regions such as IPS [38,39]. The Corsi blocks task is a well-studied behavioural working memory task in which participants observe a person pointing to a sequence of spatial locations and then must point to the same locations in turn [40]. The traditional Corsi blocks task confounds visuospatial working memory and imitation learning, but a version of the task modified for use in the fMRI environment which included no biological demonstration found activation in posterior parietal cortex during the encoding of items’ spatial locations [41].

### Differences between Hand and Text demonstrations

Another of our aims in the present paper was to examine neural regions involved in sequence imitation; it is important to know if these regions respond only to imitation, or are also engaged by other types of learning. Classic neurocognitive models of imitation imply a dedicated imitation system instantiated in the human mirror neuron system [42]. Such a system would be revealed in the contrast between observing demonstrations which can be imitated (Hand-cognitive and Hand-spatial conditions) and observing demonstrations which cannot be imitated (Text-cognitive and Text-spatial conditions). We would expect that these areas should be engaged in both the HS > TS and HC > TC contrasts (Fig 2). In our data, however, only one brain region meets these criteria. The middle intraparietal sulcus (Fig 7B) was engaged when both the cognitive and spatial tasks were demonstrated by a hand, but not for the same sequences when instructed by text. However, this area did not show a greater response to Hand demonstrations compared to Ghost demonstrations, so it is not possible to support a strong claim that this region is for imitation if operationalised as copying specific bodily movements [43]. Instead, this region appears to be critical for vicarious learning more generally.

Searching beyond the traditional mirror system areas, we find that lateral occipital regions are strongly activated for viewing Hand demonstrations compared to Text or Ghost demonstrations (Fig 6) and are active across both cognitive and spatial tasks. These regions could be responding only to the visual features of the hand, as found in previous studies of the observation of hand actions [44].

Overall, we found two areas that were more active for Hand than Text demonstrations: middle IPS and lateral occipital cortex. However, these differences could be due to specialisation for tasks other than imitation, and we consider these more in the section below.

### Differences between Hand and Ghost demonstrations

To understand the specificity of imitation learning, it is important to compare how people learn by imitation to how they engage in other forms of learning. Ghost control conditions are commonly used in developmental studies, to contrast the imitation of actions against the emulation of a goal or the end state of an action [12]. Here we created a ghost control where participants saw items highlighted as in the Hand demonstration, but without a visible hand in the centre of the screen. Previous studies have demonstrated that adults and children often learn in these conditions [12]. However, both children’s [6,14] and adults’ [45] performance in these ghost learning conditions (without a model) is impoverished relative to conditions where participants witness a live model executing the target actions [46]. For example, Boutin and colleagues [45], using an arm movement serial response time (SRT) task where subjects executed complex motor-spatial sequences, demonstrated that adults in a ‘stimulus-only’ (or ghost) training condition were less likely to improve their performance with additional motor practice relative to participants in a stimulus-and-action (or video-model) condition. These results have been largely interpreted to mean that the systems mediating the coding and representation of observed responses in the ghost versus the model conditions are different.

Despite previous findings of differences at the behavioural level, few differences between Hand and Ghost conditions were apparent in the neuroimaging data in this study. Observing the Hand demonstration compared to the Ghost demonstration engaged occipitotemporal regions bilaterally for both the cognitive and spatial tasks. This result is in line with outcomes of previous studies, which show that these areas are engaged in higher-order visual processing. For instance, middle and inferior occipital cortex are engaged when viewing hands and body parts even without any motor responses [47]. There were no differences in inferior parietal or inferior frontal cortex between the Hand and Ghost conditions. This is congruent with studies suggesting that mirror neuron regions respond equally to stimuli with and without a human model if the stimuli are matched for action knowledge [48].

### Is there evidence for task-specific systems dedicated to imitation?

Developmental data showing that children can perform cognitive imitation tasks before spatial imitation tasks might be taken to imply that there are specialised systems in the brain to enable cognitive imitation [13,49,50]. Such systems would be revealed by interaction contrasts between task and demonstration type. We found just one such interaction, in bilateral inferior frontal gyrus (Fig 8). This area was more strongly engaged by the Hand demonstration of the cognitive task than by the equivalent demonstration for the spatial task. This suggests that IFG activation could be specific to cognitive imitation. However, we note that this area did not distinguish between Hand and Ghost demonstrations, suggesting that this region may be more generally attuned to vicarious input (social and non-social alike).

To place this in the context of previous findings, many studies have linked IFG to imitation [17]. IFG is an area which we hypothesised would have a role in imitation a priori, because it has traditionally been considered part of the ‘mirror neuron system’ and is a strong candidate region for an imitation area [51]. Previous data also show a role for IFG in goal-directed action imitation [52]. Our data suggest that this imitation area is specific to object-directed actions, and is not engaged when participants observe action sequences to particular spatial locations.

Overall, our data suggest three regions within the classic mirror system which are engaged in our imitation tasks: the IFG, middle IPS, and anterior IPS. We suggest that IFG and middle IPS work together in the Hand-cognitive task, while anterior IPS and middle IPS work together in the Hand-spatial task. The finding that there is no single brain network involved in imitation tasks is consistent with prior fMRI studies showing that the entire MNS network is not active for every imitation task [17,20,24].

Novel imitation (i.e., the learning and copying of novel responses) may be distinct from familiar imitation (i.e., the recall of previously learned responses); in this study, participants engaged in novel imitation, as sequences were unique on each trial. While the observation and execution of familiar actions engages canonical MNS regions [48,53], the imitation of novel responses does not always do so [16]. We are not the first to point this out [54]. Some novel tasks that involve multiple actions likely require copying both object-specific (i.e., cognitive imitation) and spatial-specific (i.e., spatial imitation) responses simultaneously. Perhaps not surprisingly, these more complex tasks engage both IPS and IFG [48,55].

### Is this imitation?

The tasks we use in the present study are closely modelled on our developmental research tasks, but differ from other tasks commonly used in fMRI studies of immediate imitation. Thus, some might argue that our tasks do not tap ‘imitation’ as typically studied in cognitive neuroscience. Following the conventions of the behavioural sciences, we have operationalised imitation as the copying of a novel sequence [45,56–58]; resulting in the learning of a ‘novel or otherwise improbable act’ [59]: p. 122. As Heyes [60] has noted, what makes a task a social learning task—as opposed to an individual learning task—is the source of the input: another agent versus oneself. To evaluate imitation learning specifically, it is necessary to evaluate whether the observer (a) learned from and (b) replicated the observed event. In our Hand demonstration conditions, the correct sequence for the spatial and cognitive tasks was demonstrated by another person, giving participants the opportunity to imitate.

It is important to consider that participants might have used other strategies. For example, it is possible for participants to solve the Hand-cognitive task by ignoring the central video clip of the hand operating the joystick, attending only to the identities of the objects in each sequence, and converting them to a subvocal list. The present study controlled for this possibility using the Text instruction condition. If subjects used a subvocal list strategy in each condition, then there should be few or no significant differences between Hand and Text conditions. Yet there were robust activation differences between the Hand and Text conditions for both tasks (Fig 6). Thus, we believe our study provides a valid and useful investigation of the neural systems of cognitive and spatial imitation.

### Comparisons to children’s performance

An aim of the present study was to use studies of sequence imitation in children to inform our characterisation of the neural systems of imitation in the adult brain. This means it is important to consider how behaviour on this task compares in children and adults. Adult participants in this study were slower to select sequences in the cognitive task than the spatial task, and they experienced greater error rates when doing so. Slower and more error-prone performance indicates that the cognitive task may have been somewhat more difficult for adults than the spatial task. These behavioural results are in contrast to preschool children’s performance, which is less accurate when imitating in the spatial task than the cognitive task. Additionally, children’s performance is similar (if slightly less accurate) when given ghost demonstrations of the cognitive task compared to social demonstrations (imitation) [14]. In the present study, adults’ accuracy as measured by error rates was also similar in the Ghost and Hand conditions, indicating that their ability to learn sequences in the emulation condition is unimpaired.

Consistent with behavioural differences observed when children imitated in the cognitive versus the spatial task, in the present study with adults, differences emerged in brain involvement between the tasks. Specifically, several brain areas (fusiform gyrus, IPS) showed consistent activation differences when observing demonstrations of the two tasks (Figs 4 and 5). Future neuroimaging studies with children would help to clarify whether the observed differences in children’s behavioural performance in these tasks has to do with delayed maturation of IPS associated with imitation performance in the spatial task relative to the maturation of inferior frontal areas associated with imitation performance in the cognitive task. In fact, various large-scale neuroimaging studies have shown delayed maturation of parietal areas relative to frontal areas (for a review see [61]). However, how the maturation of these cortical regions alone and their connections with other neural structures affect imitation performance remains an open question.

### Limitations

The present study focuses only on the demonstration phase of the task, rather than the subsequent performance of imitation. This is because the richness of the present data leaves little space to discuss further results. Recent research indicates that observing with the intention of imitating an action (i.e., a delayed imitation task) and passively observing an action with no intention of imitating activate different brain regions, including inferior parietal lobule and parts of premotor cortex [62]. Further, observing a video of a hand action with the intent to imitate the action and observing the same video with the intent to judge the action’s velocity activate different brain regions; for the imitation intention, inferior parietal, premotor, and inferior frontal cortex are all differentially engaged [63]. Thus, observation of a demonstration with the intent to imitate it is an important component in the imitation process.

It is possible that for adults performing the cognitive task, the spatial information from the observation phase interferes with the correct selection of items in the execution phase, resulting in less accurate performance than in the spatial task. However, if this is the case for adults, it is not the case for young children, as 3-year-olds’ imitation performance is more accurate in the cognitive task.

Finally, the differences between conditions were subtle. Though participants could have adopted similar sequencing strategies for both the cognitive and spatial tasks and for the different demonstration conditions (such as relying mostly on subvocal repetitions of the items’ order), the finding of robust activation differences between tasks and demonstrations shows that they did not. It remains possible that larger differences in brain activation could be found if our tasks and demonstrations differed more, but in such a study it would also be harder to interpret the results, in which case larger differences in brain activation could simply be due to the corresponding differences between the tasks. Our closely matched stimuli provide precise control of the task and the level of social information in each trial, and thus provide a strong test of the brain systems for sequenced imitation behaviour.

## Conclusions

Much remains to be explored regarding how people learn new things from others and how different brain areas work together to achieve this. After controlling for language and working memory (Text condition), we find two distinct brain regions used in imitation learning. Specifically, observing to imitate novel object-based rules (cognitive imitation) from a Hand demonstration selectively activated IFG, while observing to imitate novel location-based rules (spatial imitation) from a Hand demonstration activated left anterior and middle IPS. These results indicate that non-overlapping brain systems are involved in the imitation of different types of sequences, and may help to explain differential imitation performance in these tasks in children. Future work can trace how these regions change in typical and atypical development, and how the neural systems underlying these two exemplars of imitation relate to other forms of imitation behaviour.

## Supporting information

S1 Table Regressors used for analysis. (XLSX)
